# Protective Efficacy of BCG Vaccine against *Mycobacterium leprae* and Non-Tuberculous Mycobacterial Infections

**DOI:** 10.3390/vaccines10030390

**Published:** 2022-03-03

**Authors:** Davit Orujyan, William Narinyan, Subhapradha Rangarajan, Patrida Rangchaikul, Chaya Prasad, Beatrice Saviola, Vishwanath Venketaraman

**Affiliations:** College of Osteopathic Medicine of the Pacific, Western University of Health Sciences, Pomona, CA 91766, USA; davit.orujyan@westernu.edu (D.O.); william.narinyan@westernu.edu (W.N.); subhaprad.rangarajan@westernu.edu (S.R.); patrida.rangchaikul@westernu.edu (P.R.); cprasad@westernu.edu (C.P.); bsaviola@westernu.edu (B.S.)

**Keywords:** non-tuberculous mycobacteria, BCG, *M. leprae*, *M. abscessus*, *M. marinum*, *M. avium*, *M. ulcerans*

## Abstract

The genus mycobacterium includes several species that are known to cause infections in humans. The microorganisms are classified into tuberculous and non-tuberculous based on their morphological characteristics, defined by the dynamic relationship between the host defenses and the infectious agent. Non-tuberculous mycobacteria (NTM) include all the species of mycobacterium other than the ones that cause tuberculosis (TB). The group of NTM contains almost 200 different species and they are found in soil, water, animals—both domestic and wild—milk and food products, and from plumbed water resources such as sewers and showerhead sprays. A systematic review of Medline between 1946 and 2014 showed an 81% decline in TB incidence rates with a simultaneous 94% increase in infections caused by NTM. Prevalence of infections due to NTM has increased relative to infections caused by TB owing to the stringent prevention and control programs in Western countries such as the USA and Canada. While the spread of typical mycobacterial infections such as TB and leprosy involves human contact, NTM seem to spread easily from the environment without the risk of acquiring from a human contact except in the case of *M. abscessus* in patients with cystic fibrosis, where human transmission as well as transmission through fomites and aerosols has been recorded. NTM are opportunistic in their infectious processes, making immunocompromised individuals such as those with other systemic infections such as HIV, immunodeficiencies, pulmonary disease, or usage of medications such as long-term corticosteroids/TNF-α inhibitors more susceptible. This review provides insight on pathogenesis, treatment, and BCG vaccine efficacy against *M. leprae* and some important NTM infections.

## 1. Introduction

The species of bacteria in the genus mycobacterium are known to cause a variety of infections in humans. Due to the interplay of the host defense and the infectious agent, these infections are classified as *M. tuberculosis* complex (MTC) or non-tuberculous mycobacteria (NTM).

Non-tuberculous mycobacteria include all the species of mycobacterium other than the ones that cause TB or leprosy [1]. *Mycobacterium leprae,* a non-motile, acid-fast bacillus from the mycobacterium family, is a non-culturable, obligate intracellular pathogen that causes leprosy, a chronic granulomatous infection characterized predominantly by peripheral nerve damage and prominent skin lesions.

According to Runyon classification, NTM are further classified according to their growth rates as slow growing mycobacteria, SGM (types I, II, III), which take more than 7 days, and rapid growing mycobacteria, RGM (type IV), which takes less than 7 days. Within SGM, each type is defined by its ability to produce pigment. Type I can only produce a yellow pigment in the presence of sunlight, i.e., photochromogen, type II can produce pigments irrespective of the presence of light, i.e., scotochromogen, and type III produce very little or no pigmentation, i.e., achromogen. Type IV or RGM is not associated with the characteristic of pigmentation [2,3].

The infections caused by these NTM can be from about 200 different species and are predominantly found in the environment and animals [4]. Although the incidence of TB has declined, an inverse relationship has been observed between TB and NTM infections, with the rates of NTM infections showing a simultaneous and significant increase worldwide within the past 70 years [5].

As a result of the control programs implemented by Western countries such as the USA and Canada, infections due to the MTC are significantly less than those caused by NTM [6]. Due to the prevalence of NTM existing in the environment, the infecting agent has the ability to spread much easier when compared to infections causing TB and leprosy, which involve more human contact [7]. As a result, it makes these NTM highly opportunistic, which increases their chance of infecting the immunocompromised including individuals with HIV, genetic immunodeficiencies, or acquired decrease in their immune system [3,8].

As opposed to MTC that cause TB and leprosy, NTM have been shown to be less virulent [9], although *M*. *abscessus* is known to be one of the most drug-resistant of all mycobacteria. *M. avium-intracellular* complex are common NTM that can cause active pulmonary and extra pulmonary disease. Other NTM that are commonly associated with skin infections are *M. ulcerans* and *M. abscessus*.

Bacillus Calmette–Guérin (BCG) is a vaccine made from attenuated strains of *M. bovis*, a close relative of *M. tuberculosis,* and is routinely used in countries where TB is hyper-endemic. While it is hitherto not used in the Western countries owing to the lower incidence of TB, and the varying effectiveness of the vaccine against TB, it might be of use in preventing NTM infections. It is the most widely administered vaccine and usually a part of the routine newborn immunization schedule. BCG vaccine also offers partial protection against non-tuberculous mycobacterial infections like leprosy and Buruli ulcer [10]. Calmette and Guérin began their research in 1900 for an antituberculosis vaccine. After more than a decade of attempts to develop a vaccine for TB, they found success in using an attenuated *M. bovis* strain. In 1919 they attempted a vaccination trial using guinea pigs, rabbits, cattle, and horses. They were successful in preventing the vaccine subjects from contracting progressive TB [11]. Thus, in 1921 they decided it was time for trials involving human subjects for the vaccine. The vaccine was given via oral route to infants at the Charité Hospital in Paris, and later it was concluded that there was a decrease in TB mortality among the infants that were given the BCG vaccine. As a result, BCG vaccination spread to various countries [10,11]. A probable protective effect of BCG vaccine against NTM infections could be drawn from a nationwide surveillance study conducted in Sweden after it discontinued general BCG vaccination of newborns in 1975. Annual incidence rate of NTM infections per 100,000 children less than 5 years of age increased from 0.06 between 1969 and 1974 to 5.7 during 1981–1985. The cumulative incidence rate of NTM infection per 100,000 children less than 5 years of age between 1975 and 1985 was estimated to be 26.8 among non-BCG vaccinated children, and 4.6 among BCG vaccinated [12]. Mycobacterial cervical adenitis, caused by a NTM infection, was an uncommon disease in Finland from 1977–1986, where neonatal BCG vaccination was in practice, with an incidence rate of 0.3 per 100,000 children. Contrast that with that in Sweden, where the BCG vaccination had been discontinued, and where the incidence was 30 times higher [13].

It is well known that immunodeficiency caused by HIV infection leads to many opportunistic infections. With increasing life expectancy of HIV-infected patients with the advent of combined anti-retroviral therapy, opportunistic infections are managed through prophylactic pharmacotherapy or preventative measures. Pulmonary infections with NTM are more prevalent in people living with HIV, probably due to the impaired T cell-mediated immunity. However, robust diagnostic guidelines for pulmonary infections by NTM in people living with HIV are yet to be established. In a retrospective study using data from an HIV-associated pneumonia management program at Louisiana State University, between 2007 and 2011, 96 out of the 196 study subjects tested positive for NTM of undetermined significance and 73 tested positive for pathogenic NTM, with MAC being the most frequent. This underscores the importance of establishing a uniform and regular diagnostic protocol for NTM in patients infected with HIV [14].

An approach to clearly delineate NTM diagnoses will also be helpful in differentiating their presence from that of TB, as seen in another systematic review analyzing the relative proportion of NTM infections versus TB at death among HIV patients in sub-Saharan Africa, which showed that for every seven HIV patients who died with mycobacterial infections, one died with NTM infection. In the absence of prophylaxis and diagnostic criteria for NTM, pulmonary infections in patients with HIV infections could be mistakenly treated for TB instead owing to their shared clinical features. Five patients with NTM infections on postmortem culture did not have culture evidence for TB [15].

A positive correlation between an immunocompromised state and COVID-19 infection has been reasonably well established. BCG has been shown to modulate innate immunity called ‘trained innate immunity’, in which activated innate immune cells such as monocytes, macrophages, and NK cells create an altered and improved immune response to challenges posed by unrelated stimuli. Countries that had a mandatory BCG vaccination approach have had lower COVID-19 infections and death rates; however, further research is needed to eliminate any confounding factors and establish a direct correlation. Clinical trials are currently being conducted in countries such as Australia, Netherlands, and the USA to determine the protective effects of BCG against COVID-19 [16].

The current debate as to whether BCG vaccine is effective against TB has been going on for many decades. In the 1950s, the variation in its results became apparent with a UK trial showing more than 75% protection [17], while one in the USA [18,19,20,21] and Puerto Rico [22,23] demonstrated less than 30% protection. One of the hypotheses first put forth by Palmer et al. was that BCG exhibited higher efficacy in populations with lower exposure to atypical mycobacteria, such as those far away from the equator, than in those populations in the tropics with higher exposure to NTM [24]. Fine et al. finetuned Palmer’s hypothesis into one that classified efficacy of BCG vaccine against tuberculosis (TB) by latitude [25]. An effort to explain such variances based on latitude was carried out in a meta-analysis by Wilson et al. Their study included factors such socioeconomic status, climate, storage of vaccine, population density, baseline health of the population, etc., as plausible influencers but concluded with a need for robust scientific data to draw any correlation [26]. Another meta-analysis done by Zodpey and Shrikande including 80 studies across the world showed a statistically significant correlation between latitude and protective effect of BCG in clinical trials, but not so for observational studies. According to this analysis, latitude accounted for 15% of the variance observed in the protective effect of the BCG vaccine [27]. Such a variance could possibly explain, at least to some extent, the failure of a BCG vaccination clinical trial conducted in a rural community of Chingleput in the state of Tamil Nadu in south India. An extensive exploration of possible factors for the BCG vaccine’s failure to elicit protection against TB after 7.5 years of follow-up failed to distill the critical factors. One possible explanation posited was that BCG might be protective against endogenous reactivation but not against exogeneous reinfection, given that among the study population, the prevalence of TB was high in the middle-aged and elderly men, with newly infected persons developing the disease less frequently. In the absence of any protective effect by BCG vaccine in this trial, the hypothesis that an innate immunity already developed in the population on account of exposure to atypical mycobacteria masking any protective effect of BCG vaccine could not be explored further [28]. Similar findings resulted in a 15-year follow-up of the trial [29]. A clinical trial comparing the cell-mediated immune response through assessment of lymphocyte proliferation, and IFN-γ release before and after BCG vaccination between patients that were initially PPD positive and PPD-negative individuals, demonstrated no effect of BCG in driving the immune response as a protective measure [30].

The tuberculin skin test (TST) has been in use since 1907, when Von Pirquet first introduced it using a reagent that was a mixture of proteins and other macromolecules derived from tubercle bacillus. Since then, standardization of the reagent’s contents has been a constant endeavor. The first reference to such a reagent as purified protein derivative (PPD) was in 1934, when a biochemist at the Henry Phipps Institute at the University of Pennsylvania, Florence B. Seibert, created a reagent that was relatively less in other macromolecules and rich in proteins from *M. tuberculosis*. It was then renamed as PPD-Standard or PPD-S and adopted as the international standard by the WHO in 1952. An international unit for PPD was then defined as the biological activity contained in 0.028 μg of PPD-S. Several other PPD formulations are in use across the world apart from in the USA and Canada. One of them, PPD-RT23 SSI, is the most widely used to estimate the prevalence of tuberculosis in most of the endemic countries including India, Yemen, and South Africa. Some of the other PPD formulations in use are PPD RT 23 Mexico, PPD-s, and PPD IC-65 [31]. A study conducted by Schiller et al. comparing IFN-γ release in whole blood samples of cattle stimulated with different bovine tuberculin preparations demonstrated significant differences in responses [32]. Such variation in responses calls for a robust analysis of each PPD’s components. There have been some studies with proteomic analyses of different PPDs, which portray component proteins that are conserved among most mycobacterial species [31]. Such overlap might explain the large cases of false positives with TST. The specificity of TST as a diagnostic tool for tuberculosis could potentially be enhanced if the components are standardized across the globe and contained only those that are unique to *M. tuberculosis*.

A topic that warrants discussion is the interaction of the BCG vaccination and the presence of NTM in the environment. The BCG vaccine is known for having lower efficacy for full protection against respiratory TB and, over the past few decades, many attempts have been made to find methods of boosting the BCG vaccine [33]. Evidence has been shown that the BCG vaccine’s efficacy is directly related to the geographical location of where the vaccine was administered and there is still much to understand regarding why exactly this is the case [34].

Poyntz et al. proposed that an increased exposure to NTM after BCG vaccination may play a role in this location-dependent variation. A murine model was utilized to administer either live (oral) or killed (systemic) *Mycobacterium avium* in those with BCG vaccination. The findings showed increased Th1 and Th17 responses in those exposed to killed *M. avium*, which is associated with increased protection. On the other hand, those exposed to live *M. avium* showed increased levels of Th2, associated with decreased protection, thus coming to the conclusion that exposure to NTM may induce varying effects on BCG vaccine efficacy, depending on route and viability [35]. With this knowledge, we are aware of the need for better models to understand exactly how different NTM exposure conditions may compromise future forms of the BCG vaccine.

Although the BCG vaccine is used mainly for the prevention of TB, in this review we will discuss the efficacy of the BCG vaccine, along with other possible vaccines, against the non-tuberculous mycobacterial skin infections highlighted below, as well as their presentation, pathogenesis, diagnostic methods, and treatment options.

## 2. *Mycobacterium leprae*

*Mycobacterium leprae* is a non-motile, acid-fast bacillus from the mycobacterium family [36]. *M. leprae* is a non-culturable, obligate intracellular pathogen that causes a chronic granulomatous infection characterized predominantly by peripheral nerve damage and prominent skin lesions known as Leprosy or Hansen’s disease [37]. Endemic mostly to tropical underdeveloped and developing countries, most commonly Brazil and India, *M. leprae* is transmitted mainly by entry through the nasal mucosa into the upper airway, which constitutes one of the most important entry routes [38,39,40,41]. *M. leprae* is found within environmental soil and water and is zoonotic with a natural host most commonly being the nine-banded armadillo [39]. *M. leprae* has a slow doubling time of 12 days, and thus in its early stages of infection is not highly contagious. Clinical diagnosis of leprosy is based on the manifestation of skin lesions with associated sensory loss and can be made primarily through skin biopsy, but also it includes serological and polymerase chain reaction tests [42,43]. In its early or indeterminate stages, leprosy is characterized by poorly demarcated borders and hypopigmented macules. Furthermore, in its determinate stages, leprosy presents with various histopathological manifestations that are dependent upon cellular responses towards the pathogen. Based on the Ridley–Jopling system, leprosy has been classified into the following categories based on the Ridley–Jopling classification: tuberculoid (TT), borderline tuberculoid (BT), mid-borderline (BB), borderline lepromatous (BL), lepromatous (LL), and indeterminate (I). Individuals who are immunocompetent present with the tuberculoid form, also known as paucibacillary leprosy; individuals who are immunocompromised present with the lepromatous form, also known as multibacillary leprosy [36,42,44,45]. Additionally, lepromatous leprosy patients are also at risk of developing type 1 (T1R) and type II reactions (T2R). T1Rs or reversal reactions (RR) are inflammatory exacerbations of the skin lesions and nerve trunks, resulting in sensory and motor alterations. T2Rs are characterized as acute with systemic involvement, also known as erythema nodosum leprosum (ENL). Leprosy is an important global health concern [46]. Contrary to popular folklore, leprosy is not highly contagious, and effective treatment is available.

### 2.1. Pathogenesis and Etiology

Infection of peripheral nerves by *M. leprae* is a hallmark of leprous neuropathy, causing sensory, motor, and autonomic disability, thus making it one of the most common causes of peripheral neuropathy worldwide [47,48]. Although *M. leprae* has a strong predilection for Schwann cells of peripheral nerves, it also infects histiocytes and keratinocytes [37]. Normally, upon pathogenic infection, antigen-presenting host dendritic cells (DC) phagocytose the pathogen and present its antigen on major histocompatibility (MHC) complexes class I and class II to T cells, which then trigger cell-mediated immune responses towards the pathogen. Although individuals affected with paucibacillary leprosy present with skin and nerve lesions, T cells in these individuals act to localize bacterial spread, thereby limiting dissemination of the disease. However, in those affected with multibacillary leprosy, cell-mediated responses are not elicited sufficiently, leading to more severe manifestations of leprosy [49]. Furthermore, in vitro analysis of *M. leprae* in the presence of human peripheral blood cells shows that antigen presentation via MHC I and II was downregulated, with greater downregulation associated with a greater inoculated dose of *M. leprae.* As a result, *M. leprae*-infected DCs and macrophages are not able to strongly stimulate CD4+ and CD8+ T cells, thus compromising host defenses against *M. leprae,* which are primarily mediated by interferon gamma (IFN-γ) secreted by cytotoxic T cells [50,51]. In addition, *M. leprae* has been shown to elicit decreased production of proinflammatory cytokines such as IL-6, TNF-α, IL-1β, and unremarkable levels of IL-8, IL-10, and IL-12p40 [52].

### 2.2. Vaccination

There is currently no vaccine, specific against *M. leprae*, which provides complete protection towards leprosy; however, administration of the BCG has been shown to provide some protective effects among those susceptible to infection by *M. leprae.* Although the BCG vaccine was originally intended for use against *M. tuberculosis***,** the proposed mechanism for the protective properties of BCG against *M. leprae* involve cross-reactivity B cells and T cells against mycobacterial antigens that are shared between different mycobacterial species [53]. In a randomized controlled trial conducted by Lwin et al. in Myanmar in 1985, 13,066 children aged 0–14, including 1531 children who were household contacts of leprosy patients, were inoculated with the BCG vaccine and shown to have an overall protective effect of 20.4% against *M. leprae* [54]. Fortunately, recent development of the *Mycobacterial indicus pranii* (MIP) vaccine derived from the non-pathogenic MIP has shown to improve treatment outcomes in patients affected with multidrug-resistant leprosy. MIP vaccine is an inactivated, non-tuberculous mycobacterial vaccine used for multibacillary leprosy patients as an adjunct immunotherapeutic agent by reducing the bacterial load and by reducing the duration of multidrug therapy in such patients by modulating the immune response towards the Th1 subtype [55,56]. In a study using guinea pig models, it was found that when the MIP vaccine was given as a booster in conjunction with the BCG vaccine, pro-inflammatory cytokines such as IL-12, IFN-γ, IL-2, IL-17, and TNF-α were increased in the infected lungs of these guinea pigs, relative to guinea pigs that were inoculated with only the BCG vaccine [57]. In addition to the MIP vaccine, the LepVax subunit vaccine based on an *M. leprae* recombinant polyprotein, which has been newly developed in the United States, has showed positive immunotherapeutic response by decreasing the neuropathic effects of *M. leprae* infection; however, testing of this vaccine in humans is currently ongoing [54].

### 2.3. Treatment and Current Research

Based on WHO guidelines, current treatment of leprosy in adults involves multidrug therapy of antibiotics. Treatment of single paucibacillary skin lesions includes a single dose of rifampicin, ofloxacin, and minocycline. Treatment of multiple paucibacillary skin lesions includes rifampicin and dapsone for six months. Treatment for multibacillary leprosy includes rifampicin, dapsone, and clofazimine for 12 months [43]. Neuritis caused by *M. leprae* must be treated aggressively to prevent or minimize nerve injury and thus prevent deformity and disability. Corticosteroids are the primary treatments suggested for neuritis and subclinical neuropathy in leprosy [58]. Use of corticosteroids and polychemotherapy to suppress the immune response are the most efficient treatment option for reversal reactions of leprosy [46]. Since *M. leprae* infects macrophages, it leads to the suppression of the vitamin D antimicrobial pathway, thus preventing the production of antimicrobial peptides, which are essential for the suppression of mycobacterial infections. As a result, supplementation of vitamin D activates the vitamin D receptor (VPR) on T cells, eliciting transformation of T cells from immature to mature. This initiates activation of the vitamin D antimicrobial pathway, which leads to the production of antimicrobial peptides, particularly cationic cathelicidins [43,51,59,60]. Another supplemental component that may aid in host defense is the use of glutathione. Glutathione is the most important endogenous tripeptide antioxidant synthesized in cells, which can exist in a reduced (GSH) or oxidized (GSSG) form. In its reduced form, GSH contains a sulfhydryl group that is involved in a plethora of reduction reactions, with its primary role being to reduce reactive oxygen species (ROS), such as peroxide and hydroxide radicals [61]. Normally, ROS produced from phagocytic cells upon pathogenic infection help damage pathogenic cells to limit the spread of infection. GSH as a reducing agent acts to reduce ROS to prevent excessive damage of host cells, thus maintaining physiological balance of ROS within the body [62]. In leprosy patients with poor immune status, severe oxidative stress has been reported because of the influence *M. leprae* has on significantly decreasing GSH levels in the body, resulting in elevated levels of ROS that will damage cellular proteins, lipids, and nucleic acids, which would ultimately lead to the progression or onset of other diseases [63,64]. To combat the rise of ROS in leprosy patients and potentially decrease the severity of disease, supplementation of GSH with N-acetylcysteine, to provide reducing equivalence, can be further researched. Furthermore, vaccination with BCG is partially protective against *M. leprae*.

## 3. *Mycobacterium avium*

*M. avium* is a non-motile, non-spore forming, Gram-positive acid-fast bacillus which inhabits soil and water environments worldwide, and along with *M. intracellular* and the newly discovered *M. chimaera,* forms the *M. avium* complex (MAC). MAC is known to be the most common NTM infection of patients with HIV/AIDS in the United States. Though *M. avium* is the most abundant species within the MAC, MAC also consists of a plethora of other mycobacterial species and accounts for nearly 80% of NTM pulmonary diseases in various countries [65,66,67]. *M. avium* itself consists of four distinct subspecies which include *M. avium* subsp. *avium, M. avium* subsp. *paratuberculosis*, *M. avium* subsp. *silvaticum*, and *M. avium* subsp. *Hominissuis,* which cause opportunistic infections in immunosuppressed patients. Individuals with pre-existing chronic pulmonary disease, such as chronic obstructive pulmonary disease (COPD), are at increased risk of MAC infection [68,69].

### 3.1. Pathogenesis

MAC is facultative intracellular and can be contracted via respiratory or intestinal routes, infecting the mucosal epithelial cells lining these systems. Once MAC crosses mucosal epithelial cells, it has a propensity to infect macrophages and monocytes, where it replicates and persists to inhibit both the innate and adaptive immune response [68]. It is believed that MAC organisms are acquired from the environment and evidence suggests that municipal water sources are an important source for MAC lung infections. Unlike with *M. tuberculosis*, human-to-human or animal-to-human transmission of MAC does not occur [65]. Host defense against MAC is primarily dependent upon natural killer cells and CD4+ T cells [70]. Normally, infected macrophages will produce IL-12, activating natural killer cells and T cells to fight the infection. However, prolonged infection with MAC leads to decreased IL-12 production, thereby suppressing host defense against *M. avium* [68]. In addition, MAC infection induces regulatory T cells (Tregs) expressing the Foxp3+ transcription factor to promote immunosuppressive cytokines, such as IL-10 [71,72]. Due to its inhibitory activity against antimycobacterial functions of macrophages, upregulation of IL-10 confers intracellular survival of MAC within host macrophages [73].

MAC infections can clinically manifest as pulmonary (patients with known pulmonary disease, without disease, solitary pulmonary nodules, and hypersensitivity pneumonitis) and disseminated (severely immunocompromised patients). Furthermore, MAC infections affecting the lungs are most common and manifest as either fibrocavitary disease in older males with a history of smoking, or nodular bronchiecstatic disease in postmenopausal women [74]. In immunocompromised patients, such as those with inherited immunodeficiency gene defects, leukemias and lymphomas, or infected with HIV/AIDS, MAC disseminates through the lymphatic system and can infect other organ systems, causing hepatosplenomegaly, osteomyelitis, septic arthritis, and generalized lymphadenitis [66,69,75]. In addition to chronic pulmonary and disseminated disease, MAC also causes skin and soft tissue infections in affected individuals, which manifest as subcutaneous abscesses, rosacea-like papulopustules, verrucous nodules, crusted ulcers, or polymorphous scaly plaques [76]. Many cases of NTM cutaneous infections are transmitted via a breach of the skin barrier such as those that occur through surgical procedures [37]. Although many disseminated diseases owing to NTM occur in immunocompromised patients, particularly those infected with HIV, patients with acquired immunodeficiency disorders resulting in high titers of IFN-γ autoantibodies are also susceptible to infection by *M. avium.* In a case report involving a previously healthy 43-year-old woman with normal CD4+ T lymphocyte levels who had underwent surgery for excision of a gradually increasing, bean-sized lump on her forehead which later resulted in acute suppurative osteomyelitis of her scalp, she later showed that she had developed disseminated infection by *M. avium* which was thereby complicated by osteomyelitis due to an acquired disorder of IFN-γ autoantibodies [77].

### 3.2. Treatment and Vaccines

Not all patients warrant treatment of MAC pulmonary disease due to the fact it is prolonged, difficult to tolerate, and only has a modest response. However, first line treatment against pulmonary MAC infection involves a series of antibiotics. Treatment for both nodular bronchiecstatic and cavitary disease includes azithromycin, rifampicin, and ethambutol three times weekly for 12 months, with the addition of amikacin for cavitary disease. In the case of disseminated disease involving lymph nodes, surgical excision of the affected nodes may be warranted [69]. In addition to multidrug therapy, the BCG vaccine has been shown to induce cross-reactive immune responses against *M. avium* in human peripheral blood mononuclear cells and mouse lymphocytes. In an experiment involving immunocompromised beige mice that were deficient in B and T lymphocytes, natural killer cells, and had reduced bactericidal activity, administration of the BCG vaccine showed reduced pulmonary bacterial burden in the lungs and spleen of these mice compared to C57BL/6 mice when both were challenged with *M. avium.* As a result, this study demonstrated that prophylactic immunization with the BCG vaccine tended to lower immunopathology within beige mice infected with *M. avium* [78]. Furthermore, the use of DNA plasmids and recombinant forms of the BCG vaccine encoding single mycobacterial genes has been shown to provide great protective effects against *M. avium* infection. A 35 kDa protein shared by both *M. leprae* and *M. avium*, but absent from the BCG, was used to develop a recombinant BCG (BCG-35) and plasmid (DNA-35) vaccine. Immunization of C57BL/6 wild-type mice by DNA-35 followed by BCG-35 was compared to that of control mice immunized with BCG alone. When immunized once with BCG-35, both groups of mice exhibited greater antigen-specific IgG titers compared to those immunized with BCG. In addition, splenocytes from mice immunized once with BCG-35 demonstrated greater proliferation and IFN-γ production. These results were similar to one-time immunization with DNA-35 alone; however, vaccination with three doses of DNA-35 yielded the most robust T cell proliferation and IFN-γ production. Although vaccination with BCG-35 resulted in more robust immune responses compared to vaccination with BCG alone, both immunization methods yielded similar reductions of *M. avium* bacterial load in the spleen of both mouse groups. Interestingly, immunization with DNA-35 alone demonstrated a more significant (2 × log_10_) reduction of *M. avium* growth [67]. Overall, the use of BCG vaccination provides prophylactic and immunotherapeutic effects against *M. avium* infection, mainly due to the induction of TB and NTM cross-reactive T cells [79]. Based on previous experimental studies, further research involving recombinant forms of the BCG vaccine that encode mycobacterial proteins shared by multiple NTM can be investigated in attempts to develop an efficacious vaccine that can induce sufficient T cell cross-reactivity between various mycobacterial species to provide the greatest immunotherapeutic effect against infection.

## 4. *Mycobacterium abscessus*

*Mycobacterium abscessus* belongs to the Runyon classification IV group of RGM [3]. Even though *M*. *abscessus* was first isolated in 1953, and a cooperative numerical phenotype study published in 1972 showed that *M*. *abscessus* was taxonomically different from *M. chelonae*, it was only in 1992 that *M*. *abscessus* was elevated to its own species status. Until then, it was considered a subspecies of *M*. *chelonae* and grouped together under the *M*. *chelonae-abscessus* complex [80,81,82]. After this taxonomic change, and with new diagnostics, it became apparent that *M*. *abscessus* was the most frequent infectious agent of all the RGM [83].

### 4.1. Characteristics

The *M*. *abscessus* complex consists of three subspecies, *M*. *abscessus* subsp. *abscessus*, *M. abscessus* subsp. *bolletii*, and *M. abscessus* subsp. *massiliense*. In addition, they exhibit phenotypic heterogeneity based on the presence (smooth variant) or absence (rough variant) of glycopeptidolipids (GPL) in the mycobacterial cell wall, contributing to its virulence. The rough variant exhibits cording, while the smooth variant exhibits sliding motility, and biofilm formation [84]. These phenotypic differences dictate the interaction of this microbe with the hosts’ macrophages, intracellular survival tactics, and its ability to make biofilms, which together decide the disease manifestations and its resistance to many chemicals, including chlorine [6,81]. The ability to spontaneously transition between smooth and rough morphologies enables *M*. *abscessus* to gain more virulence and become invasive [85,86]. Majority of isolates from lung infection by *M*. *abscessus* revealed the rough variant, while those from skin infections were found to exhibit the smooth phenotype [87]. It has been proposed that the surface glycopeptidolipids of the smooth variant are immunologically inert, and it is the loss of these molecules that results in an immunological reaction against the unmasked phosphatidyl-myo-inositol mannosides (PIMs) [84]. In some cases, expression of GPL is temperature dependent, lost at high temperatures, and is reversible at favorable temperatures [88].

### 4.2. Transmission and Pathogenesis

Transmission of *M*. *abscessus* can occur with injection of contaminated substances, use of contaminated tap water, or equipment in invasive medical procedures. Wound contamination with soil can also lead to *M*. *abscessus* infection [80,89]. It is believed to have some transmissible risk through fomites [90].

*M. abscessus* has been known to cause a range of infections including pulmonary infections in immunocompromised patients such as those with cystic fibrosis or lung transplants [6], skin infections through a variety of causes including surgeries, traumas, and contamination of injections, and other hospital-acquired infections such as those with long-term catheters, or due to the use of contaminated medical instruments, or contaminated solutions such as gentian violet [6,91]. Skin infections caused by RGM are usually deep, sometimes even leading to tenosynovitis. The localized abscesses may develop into sporotrichoid-like ascending lymphadenitis. Infections caused by *M. abscessus* seem to have a relatively worse prognosis in patients with low CD4+ T cell count [6].

*M*. *abscessus* is usually found in soil and water worldwide. In the United States, infections by *M*. *abscessus* have been mostly reported in southern states. The interactions between several components such as divalent cations, stainless steel, copper, polyvinyl chloride, and polycarbonate in potable water supplies, and the cellular composition of NTMs such as hydrophobic mycolic acid, and glycopeptidolipids have been shown to create an environment conducive to biofilm formation. RGM have been isolated from water supplies to hospital buildings [6]. Even though potable water is maintained safe through stringent processes and guidelines within the distribution system, the quality of water can fall dramatically within the plumbing of private buildings. Restricted flow creating stagnation together with temperature changes create an advantageous environment for biofilm formation [92].

### 4.3. Management of Infection

Surgical debridement becomes necessary in cutaneous infections of *M. abscessus*. Macrolides (clarithromycin and azithromycin) that bind to the 50S ribosomal subunit are primary treatment options for *M. abscessus* infections. Macrolides act as a bactericidal agent with a smaller and rapidly growing bacterial load. However, with larger loads, they tend to be bacteriostatic, probably due to poor penetration. Tigecycline and amikacin are options reserved for severe infections [6]. Presence of the *erm41* (erythromycin-resistant methylase) gene confers resistance to *M. abscessus* against macrolides [84,93]. Once transferred between bacterial cells through conjugation, the *erm41* gene integrates into bacterial chromosome. Transcription and translation of this gene produces an enzyme that methylates the 50S subunit [94]. This altered 50S subunit decreases its binding to macrolides, conferring on the cell resistance to macrolides within 3–14 days, and other antibiotics such as lincosamide and streptogramin type B [95,96].

Even though most *M. abscessus* subspecies *abscessus* and *M. abscessus* subspecies *bolletii* have an active *erm* gene, and most *M. abscessus* subspecies *massiliense* and *M. chelonae* do not, species identification alone does not seem to be enough to predict *erm41* gene activity, as mutations to the *erm41* gene could render it inactive [97,98,99]. PCR can be used to test *erm41* gene status [100], while a phenotypic drug susceptibility can confirm any activity of the gene and help tailor the treatment options. Several RGM species have an active inducible macrolide resistance gene, calling for a caution in the use of macrolides even if in vitro studies show susceptibility [101]. Amikacin and cefoxitin are a couple of common drugs used to treat *M. abscessus* complex infections [102]. Relatively new antibiotics such as clofazimine (off-label use), tedizolid, avibactam, relebactam, and vaborbactam have been shown to have anti *M. abscessus* effect in vitro [103,104].

### 4.4. Vaccination

*Mycobacterium abscessus* (MAB) infections are difficult to treat for a couple of reasons: the relatively long treatment regimen, especially in cases of pulmonary infections, and the possibility of failure and relapse rates exceeding 40%. The longer treatment plans of up to or exceeding a year put patients under continuous antibiotic exposure risk, which can potentiate further complications. Vaccination can become helpful in such scenarios by placing the focus on prevention, rather than a cure. Abate G et al. demonstrated cross protection offered by BCG against *M. abscessus* infections by showing an increase in the cytokines IL-17 and IFN-γ in by 7.2 ± 1.6 and 5.6 ± 2 pg/mL, respectively, when peripheral blood mononuclear cells from BCG-vaccinated or from latent TB-infected individuals were co-cultured with MAB-infected autologous monocytes. Cytokines IL-17 and IFN-γ are potent pro-inflammatory cytokines that play a key role in granuloma formation and subsequent immunity against mycobacterium [79].

Furthermore, the same study showed that BCG-expanded T cells inhibited intracellular MAB as potently as they did with TB, and that the CD4, CD8, and γδ subsets of T cells also inhibited intracellular MAB by 68%, 63%, and 74%, respectively. Pathogenesis of MAB infections involve intracellular proliferation within macrophages, preventing phagosome-lysosome fusion, and escaping apoptosis to further infect other cells. It is of significance to note that these subsets of T cells are keys in providing the host with immunity against any intracellular pathogens such as mycobacterium or viruses, and in potentiating the effects of cytokines such as TNF-α. Such subsets of cells are induced in greater numbers after BCG vaccination. As noted in the study, further data are needed to show the effectiveness of BCG against NTM infections in immunocompromised patients. However, given the ubiquitous presence of *M*. *abscessus* complex, its resistance to multiple common drugs, and its increasing incidence in the West, tackling it should be multifold, and vaccines should be among the front-runners in its prevention [79].

## 5. *Mycobacterium marinum*

Formerly known as *M. balnei*, *M. marinum* is a slow-growing, non-motile, non-spore-forming bacterium known for causing aquarium granuloma, or “fish tank” granuloma, infecting both humans and fish. It is a photochromogen that produces a yellow pigment when exposed to reflecting light [105]. Skin infections classically present as cutaneous sporotrichoid nodular lymphangitic lesions [106]. *M. marinum* is phylogenetically closely related to *M. ulcerans* (sharing 97% of genes), *M. haemophilum*, as well as *M. tuberculosis*, prompting the use of *M. marinum* as an attractive model for investigating TB infections [107]. Like *M. ulcerans*, *M. marinum* contains genes that allow for growth in extracellular aerobic conditions that are osmotically stable and dark [37]. They grow best at temperatures of 30 °C, and exhibit inhibited growth at temperatures above 37 °C [108].

### 5.1. Pathogenicity and Etiology

Similar to *M. ulcerans* and *M. haemophilia*, *M. marinum* cutaneous infections are often caused by freshwater or saltwater injuries, especially if the water is stagnant in nature [109]. Therefore, it most commonly affects swimmers and those who work with fish. Cases of aquarium granuloma have been reported by those working in aquariums, pet shops, preparing seafood, and more [108]. Fortunately, aquarium granuloma infections have not been seen to transmit from person to person [110]. Most common infection sites includes the dorsum of the dominant hand, as well as the elbows, fingers, and legs [111]. This is consistent with the organism having an optimum growth temperature that is cooler than body temperature [112].

The cutaneous infection presents around 2–4 weeks post-injury as either a purple nodule or a plaque at the trauma site and is often painful. It may become ulcerated or crusted over time [113]. In more severe cases, there may be sporotrichoid spread, as previously mentioned, where the infection moves up the arm along the lymphatic pathway [37]. This is more likely to occur in individuals with a compromised immune system, or those currently on a corticosteroid regimen. If the infection remains untreated and continues in chronic course, it may invade deeper tissues, infecting deeper structures such as joints and tendons. There have also been reports demonstrating *M. marinum* arthritis mimicking rheumatoid arthritis, as well as *M. marinum* causing tenosynovitis-like symptoms [114].

In terms of *M. marinum* immune response, there have been multiple studies using fish and mouse models. Hodgkinson et al. investigated the in vivo immune response in goldfish, demonstrating mycobacterial infiltrates peaking at 28 days in the kidneys and spleen, with significant increases in mRNA levels of pro-inflammatory cytokines IFN-γ, IL-12p40, and IL-1β1, as well as cytokine receptors IFNGR1-1 and TNFR2. In parallel to these changes, increases in TGF-β and IL-10 were also observed [107].

### 5.2. Diagnosis

As reported above, since aquarium granulomas have a non-specific presentation, diagnosis may be difficult based on observation alone. Often cellulitis, fungal, or parasitic infections and other skin reactions need to be ruled out first [108]. Therefore, the key to making a correct diagnosis of *M. marinum* infection is to collect a detailed history, prompting a skin biopsy, culturing of the lesion, as well as tuberculin skin testing for *M. marinum* confirmation. Both solid and broth media have been reported to be effective in culturing for *M. marinum* with solid media of Middlebrook7H11, Lowenstein–Jensen and broth medium of Middlebrook7H9, and MB/BacT. Furthermore, Lewis et al. reported that in seven patients with active *M. marinum* infection, all seven had tuberculin skin test reactions greater than or equal to 10 mm [115]. A literature review has shown tuberculin skin testing to be positive in 67% to 100% of cases. Quantiferon-TB Gold and enzyme-linked immunospot assay may show positivity but has been less helpful for diagnosis [105].

### 5.3. Current Treatment and Prevention

*M. marinum* cutaneous infections have a favorable prognosis overall. In some individuals, the infection may spontaneously resolve. However, for other patients the lesion may take up to two years to completely clear [108]. Treatment for the infection is focused on antibiotic therapy. First-line monotherapy includes a course of minocycline, trimethoprim-sulfamethoxazole, clarithromycin, or doxycycline. Antibiotic resistance is common, in which case a combination of ethambutol and rifampin have been shown to be effective. Treatment usually continues for 1–2 months after symptoms resolve, typically culminating in 3–4 months of antibiotic treatment in total [115]. Warm compresses may also be used regularly to deter the spread of infection, due to *M. marinum* showing restricted growth at higher temperatures, as previously discussed. There is also ongoing research and reports suggesting the efficacy of electrodesiccation, cryotherapy, and photodynamic therapy as effective methods of treatment when active antibiotic therapy may be complicated with other conditions that require greater attention to appropriately balance efficacy with tolerability over months of medication [108]. Wenlong et al. report the successful use of 5-aminolevulinic acid-photodynamic therapy (5-ALA-PDT) combined with fractional CO_2_ laser ablation [116].

If the patient is currently on a dose of corticosteroids, it would be prudent to weigh the risks and benefits of continuing the regimen, due to evidence of immunocompromised states leading to increased risk of lymphatic spread of *M. marinum.* This would be at the discretion of the health care provider. Finally, only in severe cases would surgical debridement of the lesion be necessary to prevent more adverse outcomes [115]. In terms of prevention, workers at risk should always be advised to wear waterproof gloves when handling raw aquatic species, adopt hand hygiene policies, and protect any prior-existing skin lesions when coming in contact with still water environments, and swimming pools should be adequately chlorinated [105].

*M. marinum* itself has also been used for protection against various other mycobacterial diseases. It has been utilized as a vaccination against Buruli ulcers [117], as potential immunotherapy against *M. tuberculosis* infection [118], as well as protecting zebrafish against mycobacteriosis [119]. Tian et al. showed that a *M. marinum* co-culture group exhibited increased expression of CD209, CD68, CD80, and CD86 than the BCG and *M. tuberculosis* groups, as well as increased IL-1β, CXCL10, CXCL8, and TNF-α [118].

### 5.4. Vaccination

In reference to using the BCG vaccine against *M. marinum* specifically, there is ongoing research on improving its efficacy. One of the drawbacks of the BCG vaccine is that it contains a partial deletion of the ESX-1 type VII secretion system, which plays a crucial role in the virulence of *M. marinum*, especially in conferring sliding motility and biofilm formation. In a previous study by Lai et al., 2304 transposon mutants of *M. marinum* identified five mutants with decreased sliding motility that were found to contain mutations that interrupted the type VII secretion system ESX-1 related genes [120].

Groschel et al. discuss how recombinant heterologous BCG expression of the ESX-1 secretion system in the BCG vaccine increases cytosolic immune signaling by inducing the cGAS/STING/TBK1/IRF-3/type I interferon axis, as well as enhancing AIM2 and NLRP3 inflammasome activity, resulting in increased CD8+ effector T cells and CD4+ Th1 immune response against ESX-1 specific antigens, thereby increasing protection against *M. marinum* infection, while also maintaining low virulence [121]. This is an exciting development in further enhancing BCG vaccine efficacy.

## 6. *Mycobacterium ulcerans*

*Mycobacterium ulcerans* is a pathogen that produces Buruli ulcers, a disease of the skin and soft tissues [122]. It is named for the Buruli district in Uganda, a region where many of the early cases in the literature were described. The disease begins with the production of papules, nodules, or plaques which progress to ulcerations of the skin in humans, without the involvement of internal organs, and although it can occur anywhere on the body, most lesions are found to be on the limbs [122,123,124]. Buruli ulceration caused by *M. ulcerans* is the third most common mycobacterial disease, after TB and leprosy [125]. The bacteria is slow growing in the environment and has an incubation period of 5–8 weeks [126]. Interestingly, the ulcers produced are painless, which may contribute to patients seeking medical care in later stages [127]. *M. ulcerans* has been identified in 34 countries but is predominate in tropical rain forests, especially in West African countries, and although not proven, there is evidence that suggests it is transmitted by abraded skin or mild traumatic skin coming in contact with contaminated soil, water, and vegetation [128,129]. The World Health Organization has three categories of the Buruli ulcers based on the size of the lesion as mentioned in Table 1 below. It is found that the cytotoxic feature of *M. ulcerans* is a polyketide exotoxin, *mycolactone*, produced by a combination of three polyketide synthases and modifying-enzymes encoded by a 174-kb plasmid, *pMUM001*. This is the main potent cytotoxin produced that induces the necrosis and ulcerations seen in infected patients [130].

### 6.1. Pathogenicity

Dendritic cells are immune cells of the body, and when they are presented by a pathogen such as *M. ulcerans*, they present the pathogen’s antigens on MHC I and II to T cells. This then allows the T cells to activate macrophages to secrete cytokines such IFN-γ and TNF [131]. In mouse models, mycolactone showed evidence of stimulating cell cycle arrest in cultured L929 murine fibroblasts. In addition, intradermal inoculation of mycolactone in guinea pigs resulted in lesions similar to Buruli ulcers in humans [132]. In both mouse and human models with noncytotoxic concentrations of mycolactone, the functional and phenotypic maturation of dendritic cells were inhibited. Specifically in mouse models, it also blocked the emigration of DC from the skin to the lymph nodes. In human blood-derived DCs, it inhibited its ability to activate allogeneic T cell priming and production of inflammatory molecules [125]. In early disease states there is downregulation of the T-helper 1 cell immune response [133]. Although it only slightly changes the concentrations of IL-6, IL-12, and tumor necrosis factor alpha, it markedly reduces the concentration of IFN-γ [125,133]. The painlessness of the ulcers may also be attributed to another cellular pathway that mycolactone stimulates. It targets Angiotensin type 2 receptors, which results in potassium-dependent hyperpolarization of nerve cells, leading to its analgesic effects [134]. Like many diseases, Buruli ulcers only occur in a limited proportion of people infected by *M. ulcerans*, as healthy individuals may have specific immune responses. As such, this is further confirmation that the disease may be halted due to cellular immune response because case reports show that people co-infected with HIV have disseminated, severe *M. ulcerans* disease [133]. A complication of Buruli ulcers that prolongs wound healing is the ability of many secondary infections to act on the necrotic lesions. Superficial swabs from the Buruli ulcers have shown that some of the common bacteria found are *Staphylococcus aureus*, *Psuedomonas aeruginosa*, *Enterobacteriaceae*, and Group A/B/C *Streptococcus* [123,135].

### 6.2. Diagnosing

Diagnosing *M. ulcerans* in its pre-ulcerative stage may be difficult as you are unable to obtain samples of the bacteria, but once having the ability to acquire a sample of the bacteria using swabs and tissue specimens from the lesions, you can diagnosis using polymerase chain reaction (PCR), microscopy, and culturing [136]. However, because culturing of *M. ulcerans* takes a long time, up to about 10 weeks, the better methods which have also been shown to have very high specificities are PCR and microscopy, with PCR being the dominant method [136,137,138]. PCR works by targeting an insertion sequence (IS) element in the DNA of *M. ulcerans* obtained from the lesions, and more specifically the IS2404 is targeted for quick PCR diagnoses of Buruli ulcers [136,138,139].

### 6.3. Current Treatment

Up until 2004, the main treatment procedures for Buruli ulcers were surgical intervention to excise the ulcerative lesions in the early stages and replacement with a skin graft. However, studies have been done to provide antibiotic therapy to reduce the need for surgical excision. After many in vitro attempts with a plethora of antibiotics, the most successful was found to be a combination therapy using rifampin and streptomycin daily for 8 weeks [140]. Considering streptomycin is administered by intramuscular injections, this can cause discomfort and requires personnel to administer. Thus, further studies have been done to reduce the number of injections and provide a therapy that includes oral medications as a dominant route. It has been shown that replacing streptomycin with clarithromycin, an oral drug, in combination with rifampin can be just as effective. Treatment still includes an 8-week course of antibiotics; however, it can be done in a couple of ways. One option would be giving rifampin with streptomycin for 4 weeks, then switching to rifampin with clarithromycin for another 4 weeks. This therapy has shown to be just as effective as 8 weeks of rifampin with streptomycin. The other option would be 8 weeks of rifampin with clarithromycin. However, these latter treatment therapies have only been tested on early stages of Buruli ulcers. All treatment methods require a relatively similar amount of time to heal, which can be up to a year [141,142]. Although antimicrobial treatments have shown to be effective, depending on the stage of detection, there still exists a small proportion of cases that may require surgical intervention to either excise some of the lesions or to simply provide a skin graft; nonetheless it is used to a much lesser extent than in cases with no antibiotic treatment [140,141,142]. It has also been found that antibiotic treatment of Buruli ulcers sometimes causes a paradoxical increase in inflammatory response before the ulcers begin to heal. This phenomenon may play a part in some studies concluding treatment as a failure. The increase in inflammatory response may contribute to the assistance of immune cells and markers providing the healing process of the ulcers [140,143,144].

### 6.4. Vaccination

Currently there are no vaccinations that provide complete immunity against Buruli ulcers produced by *M. ulcerans* in humans [146]. Nonetheless, there are *M. ulcerans*-specific vaccines under study, as well as vaccines such as the BCG vaccine that provides partial immunity. Two studies were done by the Uganda Buruli Group; the first included a group of 1230 participants who tested negative for the tuberculin skin test. Out of the 1230 participants, 606 were randomized to be given the BCG vaccine and 626 participants did not receive the vaccine. The participants were examined monthly for Buruli ulcers. This first study ended prematurely, due to the participants leaving the area. A second study with a larger stable pool of participants in a different endemic part of Uganda was done in a similar manner, with the exception of tuberculin skin test results. Both studies resulted in an overall vaccine efficacy of 47%, with the highest protection during the first 6–12 months [147]. Thus, although not providing full immunity, the BCG vaccine has shown to provide some cross-protection against *M. ulcerans*. In mouse models, recombinant BCG vaccines that specifically express *M. ulcerans* Ag85A have shown to be more effective [146,147].

Furthermore, a promising new vaccine has been under study using computer software for analyses. A PE-PGRS protein was selected from which 15 T- and B-cell epitopes have been predicted. Using this, a vaccine chimera was designed by connecting these epitopes with linkers and LprG adjuvant. Using this model with computer-simulated immune responses, it showed a high level of immunoglobins, IFN-γ, and activated macrophages. These increased immune responses are the important aspect of vaccinating against *M. ulcerans* [148].

### 6.5. Future Studies

Glutathione (GSH) is a naturally occurring antioxidant in mammals to reduce ROS. Through its mechanism of reducing ROS, it has been reported to have antibiotic properties, possibly by simply allowing more immune cells to survive and continue fighting the infection [149,150]. With this in mind, it was found that mycolactone induced the production of ROS, and upregulates the gene *CHAC1*, which codes for a major glutathione degrading enzyme [149,151]. Thus, an interesting study was performed by Förster showing the effect of GSH treatment with mycolactone in vitro and the survival rates of WT cells. The study included a control group, one with the presence of 20 or 100 nM of mycolactone alone, and one with addition of 10 or 20 nM of GSH. The results showed that overall GSH increased survival rate of WT cells with mycolactone, and within the same concentration of mycolactone, an increasing amount of GSH further helped survival rates [149]. However, no research to date has been conducted on the efficacy of GSH treatment in vivo with mycolactone infections. In addition, there can be further research done including both BCG and non-BCG vaccines. Continued search for recombinant BCG vaccines that includes more specific *M. ulcerans* antigens could possibly give rise to a better immune response to protect against Buruli ulcers. Furthermore, continued experiments with the PE-PGRS protein vaccine could shed light on a *M. ulcerans*-specific vaccine.

## 7. Conclusions

**Figure 1:**
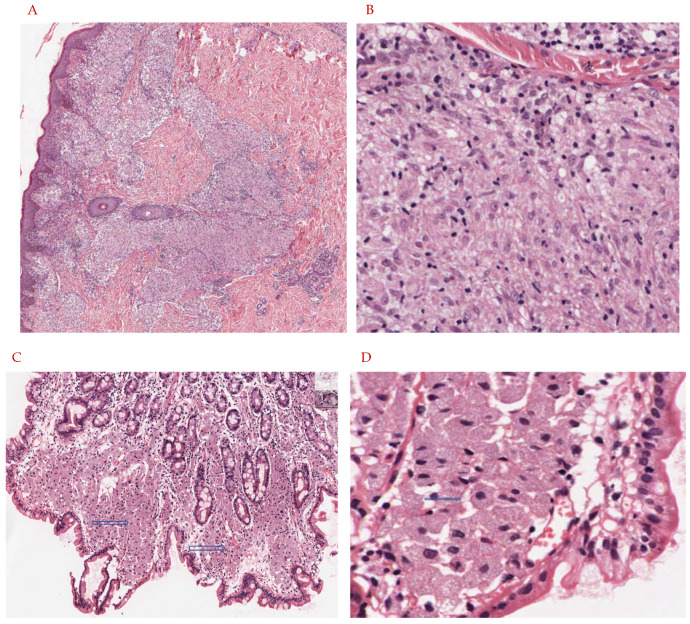

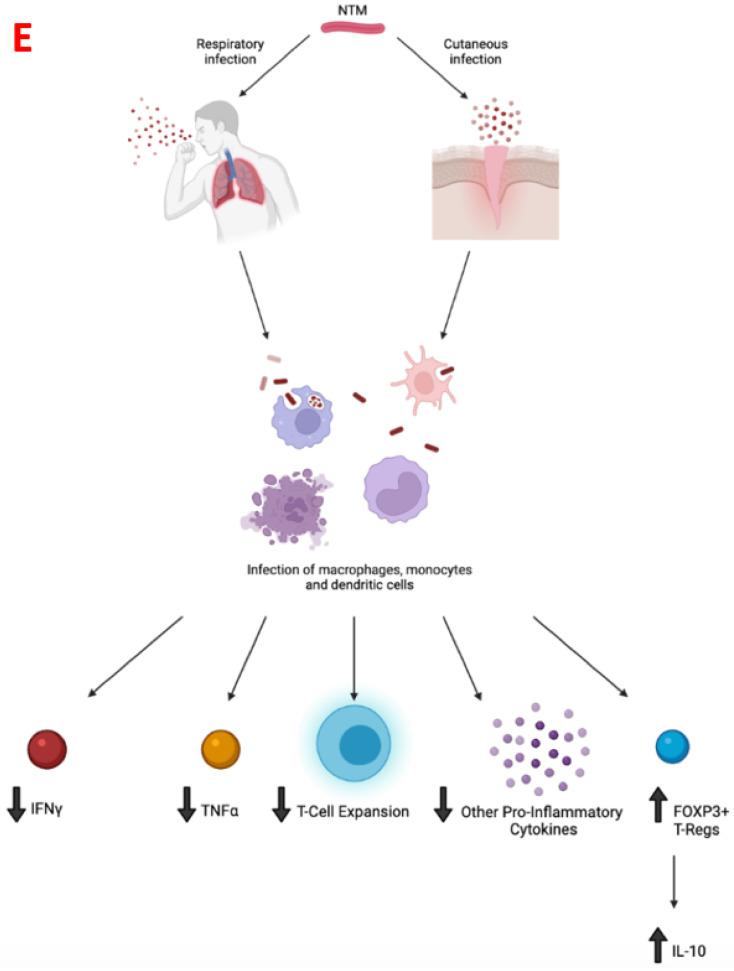
(**A**,**B**): Eleven-year-old child presents with hypopigmented annular rash of the arms, trunk, and face. The lesion is biopsied and shows an infiltrate in the papillary, mid, and deep dermis, with a suggestion of a granulomatous response. There is no evidence of caseating necrosis. On higher magnification there are nodular collections of epithelioid histiocytes with abundant eosinophilic cytoplasm. These aggregates account for the vague granulomatous response. On Ziehl Neelson stain, scarce organisms were noted, and tissue was submitted for PCR analysis. This confirmed the diagnosis of *M. leprae*. Tissue had not been submitted for cultures. (**A**): H&E stain, 20× magnification. The skin biopsy shows a vaguely granulomatous reaction in the papillary, mid, and deep dermis (blue arrow). There is no evidence of caseating or non-caseating granulomas. (**B**): H&E stain, 60× magnification. Sheets of epithelioid histiocytes are noted with abundant eosinophilic cytoplasm (blue arrow). There is no evidence of granulomas, caseating or non-caseating. There is no evidence of necrosis. (**C**,**D**): Forty-five-year-old HIV-positive patient with complaints of severe nausea and vomiting undergoes upper GI endoscopy. Lesional tissue of the small bowel is biopsied and submitted for histologic examination. Images are as noted below. The lesional tissue shows large expansions of macrophages with abundant eosinophilic cytoplasm and displaced nuclei. Granulomas, both caseating and non-caseating, were absent. There is no evidence of necrosis. On special stains (Ziehl Neelson stain) scattered organisms morphologically suggestive of mycobacteria were noted. For definitive diagnosis and species identification, tissue was sent for PCR analysis and results were consistent with *M. avium intracellulare*. Cultures were also positive. (**C**): H&E stain, 20× magnification. Small bowel mucosa shows an expansion of the lamina propria with large accumulations of foamy cells with abundant eosinophilic cytoplasm (blue arrows). There is no evidence of granulomas, caseating or non-caseating. (**D**): H&E stain, 60× magnification. Sheets of foamy macrophages with abundant eosinophilic cytoplasm (blue arrow). (**E**): Infection with NTM via inhalation of aerosolized particles into the respiratory tract or entry through a break in the skin barrier leads to infection of macrophages, monocytes, and dendritic cells and a subsequent decrease in TNF-alpha, IFN-gamma, T-cell expansion, and other pro-inflammatory cytokines thereof. In addition, upregulation of Foxp3+ and Tregs results in increased IL-10 production which promotes intracellular NTM survival within the host.

**Figure 2:**
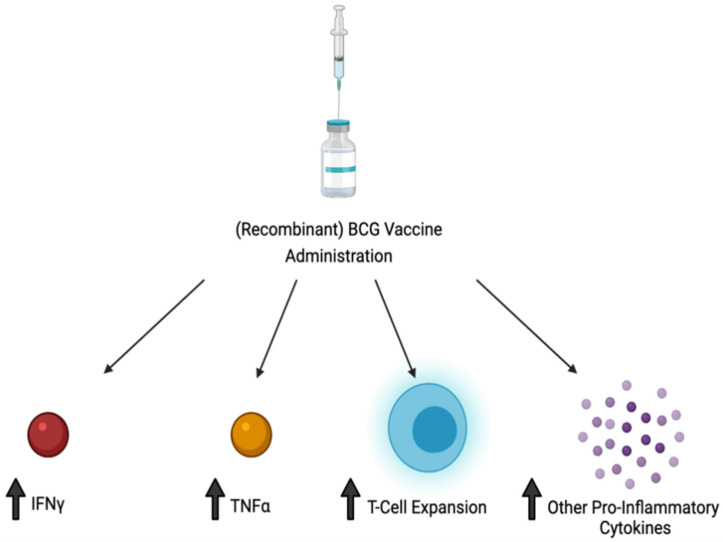
Administration of BCG and/or recombination forms of BCG result in an increase in TNF-alpha, IFN-gamma, and T cell proliferation, as well as other pro-inflammatory cytokines, leading to a more robust immune response against NTM infection.

In conclusion, *M. leprae* and many NTMs cause pulmonary and extra-pulmonary infections, with this article highlighting *M. leprae* and a handful of NTMs, of which their key characteristics are summarized in Table 2. *M. leprae* and NTMs exist in their respective environmental areas, making the contraction of the disease prevalent in both immunocompetent and immunosuppressed individuals (Figure 1A–D). When infected, these NTMs attempt to inhibit the adaptive immune response and decrease inflammatory cytokines necessary for the body to fight the infection, while the infection of some NTMs promote immunosuppressive cytokines which account for their intracellular survival (Figure 1E). As a result of the pathogenesis of these infections, immunosuppressed individuals will develop a more severe form of the disease. While these infections can range from a mild to debilitating, and sometimes fatal disease, a complete cure or prophylactic treatment is yet to be found. Most of these infections may currently have an antibiotic therapy regimen but these are mostly useful if the disease is caught in the early stages. Thus, it is important to formulate a prophylactic treatment option such as a vaccine to decrease the overall number of infected cases these bacteria cause. From the literature it is evident that the BCG vaccine, although primarily used against *M. tuberculosis*, does provide partial immunity to these NTM infections through cross-reactive immunity and results in a more robust immune response via T cell expansion and via an increase in production of pro-inflammatory cytokines (Figure 2). However, further studies can be done to increase the efficacy of the BCG/recombinant BCG vaccine or create a new vaccine to induce a more optimal immune response to fight NTM infections. Although the BCG vaccine is not currently indicated for use against NTM infections in many European countries or North America, pediatric administration should be considered early on in countries where NTM, as well as *M. tuberculosis*, are endemic. This is supported both by the rise in NTM infections of children in Sweden following the abandonment of the BCG vaccine, and by the lesser incidence of mycobacterial cervical adenitis in Finland compared to that in Sweden, as previously mentioned.

## Figures and Tables

**Table 1:** Summary of the different categories of Buruli ulcer infections according to the WHO approach and their respective treatment guidelines and primary aim of care [145].

Treatment Category	Disease Manifestation	Treatment	Primary Aim	Diagnosis
Category I	Single small lesion (i.e., nodule, papule, plaque, and ulcer <5 cm in diameter)	Complete antibiotics. If at or near a joint, maintain same movement as on unaffected side. If surgery is needed in non-critical areas, consider this after 8 weeks of antibiotic treatment	Cure without surgery. Cure without movement limitations	Clinical diagnosis with or without laboratory confirmation
Category II	Non-ulcerative and ulcerative plaque and edematous forms. Single large ulcerative lesion 5–15 cm in diameter	Complete antibiotics, before surgery if possible. If at or near a joint, maintain same movement as on unaffected side	Cure without surgery. Reduce the extent of surgical debridement when needed. Cure without movement limitations	Clinical diagnosis with or without laboratory confirmation
Category III	Lesions in the head and neck region, particularly face. Disseminated and mixed forms such osteitis, osteomyelitis, joint involvement. Multiple lesions and osteomyelitis. Extensive lesion > 15 cm	Complete antibiotics, before surgery if possible. If at or near a joint, maintain same movement as on unaffected side	Cure without surgery and without movement limitations	Clinical diagnosis with or without laboratory confirmation

**Table 2:** Summary of the various NTMs and their key characteristics regarding pathogenesis, diagnosis, treatment, and BCG vaccine efficacy.

	*M. ulcerans*	*M. leprae*	*M. abscessus*	*M. marinum*	*M. avium*
Toxin	Mycolactone	N/A	N/A	N/A	N/A
Environment	Tropical rain forest	Soil, water, 9-banded armadillos	Soil, water, or equipment	Water	Soil and water worldwide
Route of infection	Abraded skin	Respiratory	Wound contamination or intestinal	Fresh or saltwater injuries	Respiratory or intestinal
Disease manifestation	Buruli ulcers	Skin and nerve lesions	Skin infection	Skin infection	Skin lesions, fibrocavitary disease in lung, multiorgan involvement in HIV+
Pathogenesis	Inhibits DC activation of Th-1 and AGTR-2 on nerve cells	Decreases DC activation of CD4+ and CD8+ T cells	N/A	Grows in extracellular, aerobic condition	Infects and inhibits macrophages and monocytes
Optimal diagnostic method	PCR	Skin biopsy, serology, PCR	N/A	Skin biopsy and culture	N/A
Optimal treatment method	Daily rifampin and streptomycin × 8 weeks	Multidrug antibiotic therapy	Surgical debridement and macrolides	Self-limited or monotherapy with minocycline, clarithromycin, or doxycycline	Multidrug antibiotics
BCG vaccine efficacy	Mild cross-protection	Mild cross-protection	Moderate protection	Mild protection	Moderate protection (BCG-35)

## Data Availability

Not applicable.
